# Autistic and peudo-autistic traits in ongoing complex trauma

**DOI:** 10.1192/bjo.2021.644

**Published:** 2021-06-18

**Authors:** Jennifer Bryden

**Affiliations:** Royal Cornhill Hospital, NHS Grampian, Tutor with PRIME Partnerships in International Medical Education

## Abstract

**Aims:**

To compare the neurodevelopmental profiles of Albanian street children to those predicted by the Coventry grid.

**Background:**

A street children's centre had requested help to meet children's emotional needs. No program exists for children experiencing ongoing complex trauma. With input from widely-experienced consultant psychiatrist and consultant psychologist, a very low-intensity program of coping skills was piloted. Extensive anonymised notes were taken as part of the piloting.

The Coventry grid is a clinical tool comparing patterns of difficulties typically seen in autistic spectrum disorder (ASD) versus attachment difficulties. It's based on clinical experience and invites ongoing feedback.

**Method:**

12 Children aged 5–12 years completed the two-week program. The notes were examined for their relevance to areas of the Coventry Grid.

**Result:**

The children showed both traits typical of ASD and of attachment problems. Identifying emotions was impossible for the youngest group (5–7 years); while the older groups could say whether someone was likely to feel “good” or “bad” but struggled to differentiate further.

Fantasy and symbolic play were hard for the younger children. If asked to imagine a situation, they replied “but that's not happening”. One child constantly hugged a stuffed doll, but couldn't use it for play. Both younger groups found it hard to imagine a safe-place, though they could say what they wanted in it (chocolate and a working lightbulb). The oldest group all chose a real place related to the centre.

Generalising was difficult for all the children. The older children could say whether a story character was a good friend, but not apply this to real life. The youngest children were told a story about a dangerous stranger. Afterwards, the children said they would still go away with strangers as only the man in the story had said he wanted to harm children.

The younger children were diffusely attached, but the boys’ eye contact, gesturing, and language were normal in all age groups. All children formed friendships easily, played in a group and were intensely loyal to siblings. They didn't show restricted interests, distress at changes to routine or sensory difficulties. They showed good awareness of the widely divergent social rules at the centre and at home.

**Conclusion:**

The children showed a mix of traits usually associated with attachment difficulties and those usually associated with ASD. They may be different from UK clinic samples as they continued to experience severe trauma.

